# Strychnia and Morphia

**Published:** 1862-08

**Authors:** 


### STRYCHNIA AND MORPHIA.

Mr. John Horsley, analyst for the county of Gloucester, has lately drawn attention to an observation originally made by Dr. Letheby, and confirmed by Dr. Reese and Professor Thomas, of Philadelphia, that morphia has the power of disguising and destroying the colored reactions by which strychnia is recognized. His investigations have led him to the discovery of a method by which the difficulty thus arising may be overcome, and also to a new test for strychnia. He writes: "I have, however, satisfied myself of another very important fact, viz., that it need not create any alarm or uneasiness in the public mind, for the difficulty in question is more apparent than real; since, in any case where both active principles are capable of extraction from a dead body, the strychnia is effectually separated from the morphia, in a form admirably fitted for identification, by adopting the following simple method, which I had the honor of submiting to the British Association for 1856, viz., to convert the strychnia into a chromic salt, by the addition to the pure and concentrated liquid of a few grains of the neutral chromate of potash, and briskly agitating the mixture with a glass stirrer, for a minute or so, when, if strychnia be present, a more or less flocculent, but crystalline precipitate, of a golden color, will separate and collect at the bottom of the vessel. In the course of a few minutes, the supernatant liquor containing the morpha (which is much longer in precipitating), must be carefully decanted. If now a drop or two of the residue containing the golden-colored strychnian salt be taken up with a pipette, and projected into a small white porcelain dish, and touched with strong sulphuric acid, the usual purple and violet-colored indications of strychnia will be most marked. In this way, a very small quanity of the poison will suffice for numerous chemical experiments, as demonstrated by me before the Chemical Section of the British Association. A similar precipitate of morphia does not react in this way, but is turned of a green color; hence, when morphia is in excess, it has the power of completely masking the colored reaction of strychnia, which, though present, remains passive and unelicited; but, by the process described, the strychnia can be easily separated from the morphia, or any other active vegetable poison with which it may be associated, when once these have been extracted from a dead body in a sufficiently palpable form. Thus much for the method of separating strychnia when present with morphia; but beautiful and delicate as is this method of detection, yet it is very far inferior to a new reagent I have just discovered, viz., the nitroprusside of sodium, which enables me to detect the 100,000th of a grain with the greatest ease! These may be considered large figures, but are arrived at thus: One grain of strychnia is first dissolved in 100 grains of water; then one drop of this solution is mixed with 999 more of water, and a fragment of the nitroprusside of soda added. When dissolved, and well mixed by agitation, a single drop of this solution, equal to the 100.000th of the original grain of strychnia, is let fall into a small white ware dish, and, after evaporation to dryness over a steam bath, is fit for the development of the characteristic color, by merely drawing a glass rod dipped in sulphuric acid across the spot. The 3000th part only, with the best experiment, being the limit of the ordinary process, the new test, therefore, exceeds the old by 97,000 times the intensity; in other words, it is about 30 times more delicate."—*Med. Times and Gaz.*, June 28, 1862.

